# Assessing Access to Digital Services in Health Care–Underserved Communities in the United States: A Cross-Sectional Study

**DOI:** 10.1016/j.mcpdig.2023.04.004

**Published:** 2023-06-12

**Authors:** Diego F. Cuadros, Claudia M. Moreno, F. DeWolfe Miller, Ryosuke Omori, Neil J. MacKinnon

**Affiliations:** a Digital Epidemiology Laboratory, Digital Futures, University of Cincinnati, Cincinnati; b Department of Physiology and Biophysics, University of Washington School of Medicine, Seattle; c Department of Tropical Medicine and Medical Microbiology and Pharmacology, University of Hawaii, Honolulu; d Division of Bioinformatics, International Institute for Zoonosis Control, Hokkaido University, Sapporo, Japan; e Department of Population Health Sciences, Medical College of Georgia, Augusta University, Augusta

## Abstract

**Objective:**

To evaluate the spatial association between the access to broadband and social and health care vulnerability in the United States at the county level.

**Patients and Methods:**

Data from 3108 counties in the contiguous United States was used in this study. Access to broadband was defined as the percentage of population with a high-speed internet subscription. County-level data for access was obtained from the Survey and American Community Survey Geographic Estimates of Internet Use, 1997-2018. Indexes for resource-constrained health system, health care access barriers, and social vulnerability were obtained from the 2021 Surgo COVID-19 Vaccine Uptake Index and the Centers for Diseases and Control. We used spatial bivariate and multivariate analyses to determine the geospatial association between broadband access and the health care and social determinants. After identifying the geospatial clusters, their rates for the health care and social indexes were compared using generalized linear mixed-effects models.

**Results:**

We found that the United States exhibits a distinct spatial structure with defined vulnerable communities characterized by a high social vulnerability index, a high health access barrier index, and a high resource-constrained health care system index. However, we found a negative geospatial association between these 3 indexes of vulnerability and the access to broadband. We identified a geographical cluster in the southern part of the country with low broadband access and poor social and health indicators.

**Conclusions:**

Most health care–underserved communities in the United States are located in digital deserts with low high-speed internet access. These digital barriers could prevent the successful expansion of digital health care services and might exacerbate health care disparities in these vulnerable communities.

Digital health services such as telehealth have been used frequently to address gaps in health care disparities, allowing providers and patients to connect despite geographic barriers. Other benefits include optimal management of chronic diseases, shared staffing, reduced travel times, and fewer and/or shorter hospital stays.^1^ Several studies indicate that the quality of basic health care services delivered using telehealth can be comparable with that of traditional in-person services.^2^ These digital services have the potential to address some of the structural challenges for marginalized populations, such as lowering access barriers of time and distance.^2^^,^^3^ However, recent studies have raised concerns about the ability of telehealth to reduce this gap.^4^, ^5^, ^6^ In fact, telehealth coverage has worsened some preexisting health disparities among certain populations, particularly during the COVID-19 pandemic.^7^, ^8^, ^9^, ^10^ Access to digital health services cannot simply be increased without addressing potential preexisting barriers to health or shoring up public health infrastructure. Failure to address these challenges might increase the risk of perpetuating the same inequities, particularly if this digitally enabled ecosystem moves forward without proactive engagement, planning, and implementation of health equity policies.

The COVID-19 pandemic offered a landscape of the disparities in digital health access. Low uptake of video visits and barriers to access to online vaccination appointments among underserved populations^11^^,^^12^ unveiled the gaps emerging from both structural deficiencies within the digital infrastructure in the United States and a lack of attention to equity within the development and implementation of digital health services. The technical requirements to adequately access digital health services include access to a digital device, a minimum level of digital literacy, and a stable connection to the internet.^13^ Because the use of video calls is essential for the interaction between the practitioner and the patient, a broadband connection is essential to access the service. However, many Americans living mostly in rural (nonmetropolitan) communities do not have adequate broadband access and/or live in digital deserts where high-speed internet or cellphone service are unavailable.^14^ It is estimated that 21 million people in the United States lack broadband access.^15^ This digital divide has primarily affected underserved communities, such as rural populations.^16^ Access not only is limited by a lack of broadband infrastructure but other social determinants also play an important role, such as economical capacity to afford it, digital literacy, and cultural acceptance.^17^, ^18^, ^19^

Level of education, racial/ethnic origin, income, age, and access to health care services of quality are within the most important nonmedical factors that influence health outcomes.^10^^,^^20^ These factors, known as social determinants of health, account for between 30% and 55% of health outcomes and are a key driver of existent health inequalities.^21^ With the growing interest of the health care system to expand digital services, broadband access could become a key social determinant of health, which could strongly influence health disparities.^22^

As the future of digital health care service policies is designed, equity must be prioritized. An important first step is to identify which are the digitally underserved communities in the United States and quantify the access to health care services available for these communities. There is a lack of geospatial and socioeconomic information assessing the current health care needs of different communities. Geospatial analyses can provide a visual representation of data and identify spatial patterns that could assist in the strategic planning and allocation of digital and health services. The identification of vulnerable digital and health care–underserved communities would help to efficiently allocate digital and health resources toward the communities with the highest needs during this new era of telehealth.

Against this background, our purpose was to conduct data visualization and geospatial analyses to (1) determine geospatial disparities in broadband access, defining access as the percentage of the population with a subscription to high-speed internet; and (2) to assess the geospatial association between broadband access and social and health care–underserved communities in the contiguous United States at the county level.

## Methods

**Figure 1:**
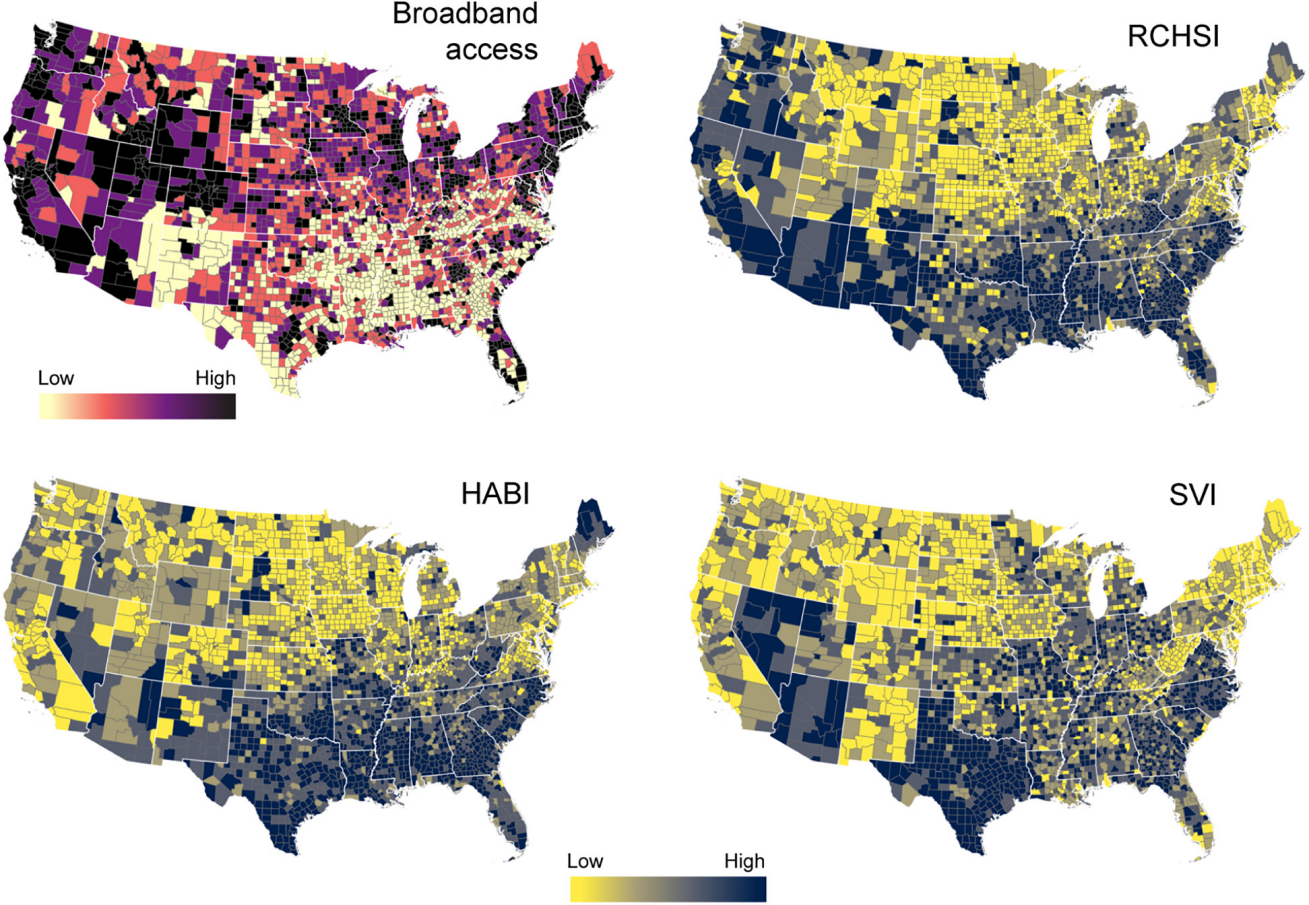
Spatial distribution of the variables included in the study, broadband access, resource-constrained health system index (RCHSI), health care accessibility barriers index (HABI), and social vulnerability index (SVI).

An institutional review board approval and informed consent were not necessary for this cross-sectional study because all data were deidentified and publicly available (Common Rule 45 CFR §46). This study follows the Strengthening the Reporting of Observational Studies in Epidemiology (STROBE) reporting guideline. We included data from 3108 counties in the contiguous US High-speed internet (broadband) access was used as the digital determinant for this study. Broadband access was defined as the percentage of the population per county that has either fixed or mobile broadband subscriptions. These data were obtained from the Current Population Survey and American Community Survey Geographic Estimates of Internet Use, 1997-2018. We included 2 health care determinants: (1) the resource-constrained health system index (RCHSI)^23^ and (2) the health care access barriers index (HABI).^23^ RCHSI is a measure that integrates indicators of low health care system capacity with indicators of health care system weakness at the county level. These indicators include health care workforce per capita, health care infrastructure per capita, health care spending per capita, and care quality indicators. HABI measures the cost and transportation factors limiting health care accessibility. It integrates measures for delayed care-seeking behavior due to cost and lack of health insurance, transportation means, and transit connectivity. High RCHSI and HABI values indicate a weak health care system capacity and low access to health care services, respectively. Counties with high RCHSI and high HABI were considered health care vulnerable. These indexes were generated for the recently released Surgo COVID-19 Vaccine Uptake Index published for 2021 (CVAC; https://vaccine.precisionforcovid.org/). We also included the social vulnerability index (SVI) published for 2020 (https://www.atsdr.cdc.gov/placeandhealth/svi/index.html)^24^ as a social determinant for our study. A high SVI value indicates high social vulnerability for that county. A detailed description of the indexes and data sources used for this study is presented in the Table, and the spatial distribution of these variables is illustrated in Figure 1.

**Table:** Summary of the Variables Included in the Analysis

Variable	Description	Source
Broadband access	Percentage of the population per county that has either fixed or mobile broadband subscription	Population Survey and American Community Survey Geographic Estimates of Internet Use, 1997-2018. https://techdatasociety.asu.edu/broadband-data-portal/dataaccess/countydata
Resource-constrained health system index 2021 (RCHSI)	This index comprises 2 subthemes using the following indicators:	Surgo Ventures—The US COVID-19 vaccine coverage index. Original source for each indicator can be found at https://cvi-data-output.s3.amazonaws.com/assets/CVAC_Methodology_Feb2021.pdf
	1. Low health care system capacity -Provider workforce per capita (total active federal and nonfederal medical doctors, doctors of osteopathy, advanced practice registered nurses, physician assistants, and pharmacists) -Infrastructure for live attenuated vaccine administration per capita (hospitals, urgent care facilities, veterans health administration medical facilities, federally qualified health centers and look-alike, pharmacies)	
	2. Weak health care system -AHRQ Prevention quality indicator -Health spending per capita -Total health care funding (CDC COVID funding, Public Health Emergency Preparedness funding, CDC grant funding for immunization and respiratory diseases and vaccines for children, and state public health funding)	
Health care accessibility barriers index 2021 (HABI)	This index comprises 2 subthemes using the following indicators:	
	1. Barriers owing to cost -Proportion of individuals without health insurance coverage -Proportion of adults who reported that there was a time in the past 12 months when they needed to see a doctor but could not because of cost	
	2. Barriers owing to transportation -Households without a vehicle -Transit connectivity index	
Social vulnerability index 2020 (SVI)	SVI indicates the relative vulnerability of every county ranking 15 social factors, including high poverty, unemployment, education, crowded housing, minority status, and disability	https://www.atsdr.cdc. gov/placeandhealth/svi/documentation/pdf/SVI2018 Documentation_01192022_1.pdf

A high RCHSI or HABI value indicates a weak health care system capacity, whereas a low value indicates a strong health care system of the county. Similarly, a high SVI value indicates a more vulnerable community.

Data visualization and geospatial analyses were implemented using a similar method applied in a previous study aimed to assess the association between the heterogeneous distribution of vaccination coverage with the dynamics of COVID-19 during the Omicron wave in the United States.^25^ In brief, the geospatial association between broadband access and the health care and social determinants at the county level was assessed using spatial bivariate and multivariate analyses in the geospatial GeoDa environment.^26^ First, spatial correlations between broadband access and each of the health and social indexes were identified using bivariate local indicators of spatial association. The bivariate local indicators of spatial association statistics identified significant spatial clustering on the basis of the degree of linear association between broadband access at a given location and the health or social indexes at neighboring locations.^27^ Maps were generated illustrating the locations with statistically significant associations and the type of spatial association between broadband access and each of the health or social indexes (ie, high-high, low-low, low-high, and high-low). Second, multivariable spatial associations between all 4 variables (broadband access, RCHSI, HABI, and SVI) were estimated using K-medians clustering analysis. K-medians is a partitioning clustering method in which the data are partitioned into *k* groups (ie, fourth groups). In this clustering method, the *n* observations are grouped into *k* clusters such that the intracluster similarity is maximized (or dissimilarity minimized) and the between-cluster similarity is minimized (or dissimilarity maximized). A further detailed description of these geospatial methods can be found in previous reports.^28^^,^^29^ After identifying the geospatial clusters, their rates for the health care and social indexes were compared using generalized linear mixed-effects models. The state was considered as a random variable to adjust for the variability generated by factors such as state health care policies and spending. Maps were generated using ArcGIS Pro,^30^ and plots were built using GraphPad Prism 9.

## Results

**Figure 2:**
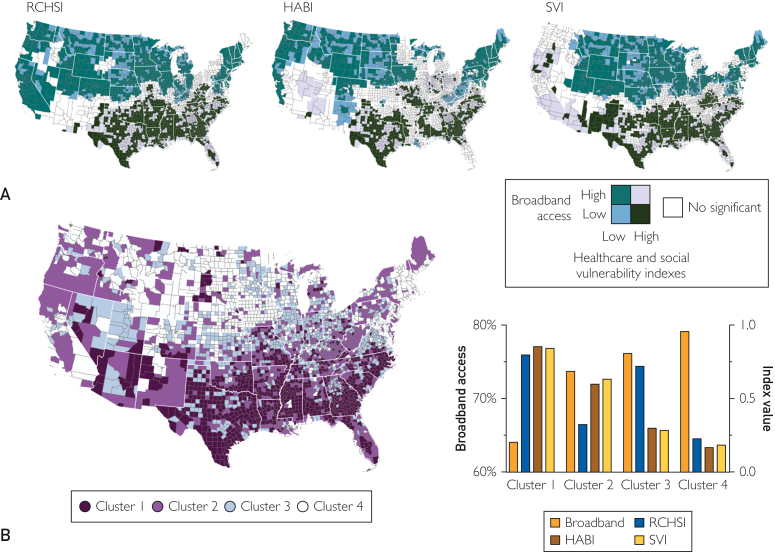
A, Bivariate spatial associations between broadband access and resource-constrained health system index (RCHSI), health care accessibility barriers index (HABI), and social vulnerability index (SVI). Dark green areas indicate counties with low broadband access and high index value associations. B, Multivariate clustering analysis including all 4 variables, with areas in dark purple (cluster 1) indicating counties with low broadband access and high values for all 3 indexes. A high RCHSI or HABI value indicates a weak health care system capacity, whereas a low value indicates a strong health care system of the county. Similarly, a high SVI value indicates a more vulnerable community.

Using bivariate clustering analyses, we identified a negative geospatial correlation between broadband access and each of the health care and social indexes evaluated. We found that the United States exhibits a heterogeneous landscape (Figure 2A-C) characterized by areas with low broadband access and high RCHSI, HABI, and SVI, which were mostly located in the southern part of the country (low-high, dark green areas in the maps); and areas with high broadband access and low RCHSI, HABI, and SVI (high-low, light green areas in the maps), located mainly in the northern part of the country. Using multivariate clustering analyses, we identified a geographically clustered area that exhibits the lowest broadband access (64%) and simultaneously the highest RCHSI (0.79), HABI (0.85), and SVI (0.84) indexes (Figure 2E; cluster 1 illustrated in dark purple areas in the map). This cluster encompassed 815 (27%) of the 3108 counties included in the study, of which 634 (78%) were rural. Broadband access was significantly higher in the areas outside of cluster 1 (76%; *P*<.001), whereas the health care and social indexes were significantly lower outside cluster 1, with an estimated median of 0.39 for RCHSI (*P*<.001), 0.37 for HABI (*P*<.001), and 0.37 for SVI (*P*<.001).

## Discussion

Our study explores the spatial association between the disparities in broadband access and health care and social vulnerability in the United States. In this study, we identified a negative geospatial correlation between broadband access and health care capacity and social vulnerability in the United States at the county level. We found that counties grouped inside cluster 1 (low-high) had the lowest broadband access (64%) accompanied by the highest health care resource constraints, the largest barriers to health care access, and the highest social vulnerability. By contrast, counties inside cluster 4 (high-low) had the highest broadband access (79%) and the strongest health care system, the lowest barriers to health care access, and the lowest social vulnerability.

Most of the vulnerable communities comprised within cluster 1 were located in the South and Southeast regions of the country, among the states of Alabama, Arkansas, Florida, Georgia, Kentucky, Louisiana, Missouri, Mississippi, North and South Carolina, Oklahoma, Tennessee, and Texas. All these states have a higher than average poverty rate with 12% to 20% of their population living in poverty.^31^ In addition, most of them are at the bottom of a ranking evaluating health care access, health care quality, and public health in the United States.^32^ Together, this highlights the deep socioeconomic and health care disparities found between the southern and northern regions of the country. The effect of these disparities has been particularly exposed by the COVID-19 pandemic. In fact, most of the southern states within cluster 1 had been at the epicenter of the different epidemic waves in the country^33^^,^^34^ and were within the top 15 states with the highest COVID-19 mortality per capita in the United States, according to the data reported by the Centers for Disease Control and Prevention (CDC).^35^ Our results show that these vulnerable areas had the lowest broadband access, which could have played an important role in the local implementation and use of telehealth during the COVID-19 pandemic.

Despite the United States being one of the countries in the world with the most robust broadband infrastructures,^36^ it is estimated that approximately 21 million people in the United States live in “digital deserts” without broadband access; of these, 14 million are rural Americans, and 1.2 million are Americans living on Tribal lands.^37^ Our study shows that 78% of the identified counties with low broadband access and simultaneous high health care and social vulnerabilities were rural. There is a critical need to target these rural communities located in the digital desert and provide the required economic resources to increase their access to broadband services. Although federal initiatives aim to decrease the digital divide, particularly for rural America, limitations still inhibit the delivery of broadband infrastructure, such as the reluctance of internet service providers to improve infrastructure and services in high-cost development areas (eg, mountainous and extremely cold regions).^22^ Federal, state, and local programs aimed at measuring and increasing broadband access should be expanded. Similarly, broadband mapping initiatives and federal-state financial partnerships should be enacted to continue identifying low-access areas and increase broadband access within those digitally underserved communities. However, although broadband and mobile connectivity are made possible across geographic divides, the economic burden of broadband services can be substantial. Hence, financial support and incentives must be made available for lower-income and underserved communities. Government subsidies for broadband access can be further expanded by relying on existing community infrastructure and social programs.^22^ For example, broadband hotspots could be set up in public spaces, such as schools, libraries, and other community centers.^38^

Digital literacy is another access barrier that needs to be prioritized, particularly when targeting rural and vulnerable communities. Telehealth use requires a minimum of digital literacy and according to data from the US Department of Education, many farmers, agricultural workers, and workers performing other rural low-skilled jobs are less likely to be digitally literate.^39^ Moreover, digital literacy also has a race/ethnicity component. Although half of the digitally illiterate population in the United States is White, when normalized to the total population per race/ethnicity, the data show those with low digital literacy are twice as likely to be Black and 3 times as likely to be Hispanic, compared with the White population. Finally, digital literacy is less common in the elderly, adding another vulnerable group to the list.^39^ The lack of digital literacy among these communities could limit their ability to obtain health care services through telehealth, exacerbating their health vulnerability. To avoid this, health care leaders and policymakers must place active collaboration across sectors to increase digital literacy within these communities. Strategies such as digital assistance in nontraditional venues such as libraries, faith-based organizations, and community groups, to help members of these communities to access and maximize the benefits that telehealth should be implemented.

Our study had limitations worth noting. An ecological study such as the one presented is an approach for examining the association between health care and other social factors at the population level. Given that, in ecological studies, it is difficult to adjust for all potential confounding factors owing to the lack of individual data, it is important to note that the reported associations do not correspond to individual risk and, thus, our results need to be interpreted with caution. Another limitation is that the most recent data set for broadband subscription available and used for this study was from 2018. Although broadband access might have increased since then, we believe this increase has followed the same spatial patterns, and thus, the spatial structure of broadband access, which is the main variable assessed in this study, might have remained constant over the years. Another limitation is that the subscription to broadband data used in this study might not provide more granular information on the underlying digital determinants, thus other data such as variables measuring digital access such as affordability, or digital literacy might be necessary to completely understand the factors driving the digital divide. Assessing health care access is a complex endeavor because it necessitates the amalgamation of diverse indicators to accurately determine the alignment between service, provider, and system characteristics and the capabilities of individuals, households, and communities. Therefore, it would be important to focus on facilitating the creation of measurement tools that more effectively capture the intricacies of access. Comprehensive analyses using consumer surveys, quality of care data, epidemiologic studies of utilization, and organizational surveys may be necessary.^40^

Health care is on the edge of a digital transformation that holds the potential to enhance health equity. However, our findings reveal that communities lacking adequate health care also tend to be digitally underserved, measured as access to high-speed internet. In light of these findings, health care providers and policymakers must incorporate strategies to bolster digital coverage, access, and usability in vulnerable communities before launching a widespread expansion of digital health care services throughout the nation. Achieving digital health care equity demands an understanding of the multifarious factors operating at multiple levels, encompassing policy, systems, community, individual, and intervention dimensions. Numerous obstacles exist in the pursuit of digital health care equity, but replicable and scalable strategies for advancing equity are also known. Examples include community co-design using inclusive principles, digital skills and literacy training with interpersonal support, and implementation strategies ensuring universal access to devices and internet connectivity. In essence, a multifaceted approach is required to accomplish digital health care equity. This entails engaging stakeholders from various sectors, fostering deep community-based collaborations, using suitable implementation methods, and adopting novel measurement approaches and standards.^10^ Similarly, the data and maps produced in this study could serve as a valuable tool in identifying digital deserts where infrastructure development is essential. By creating and evaluating tools in communities that need and can gain from digital health care services, we can better ensure that the digital health care revolution benefits everyone, leaving no one behind.

## Potential Competing Interests

The authors report no competing interests.
